# Supplementary figures and images for: Constructing temporal regulatory cascades in the context of development and cell differentiation
Source: PLoS One. 2020 Apr 10;15(4):e0231326. doi: 10.1371/journal.pone.0231326 (PMC7147753; doi:10.1371/journal.pone.0231326)

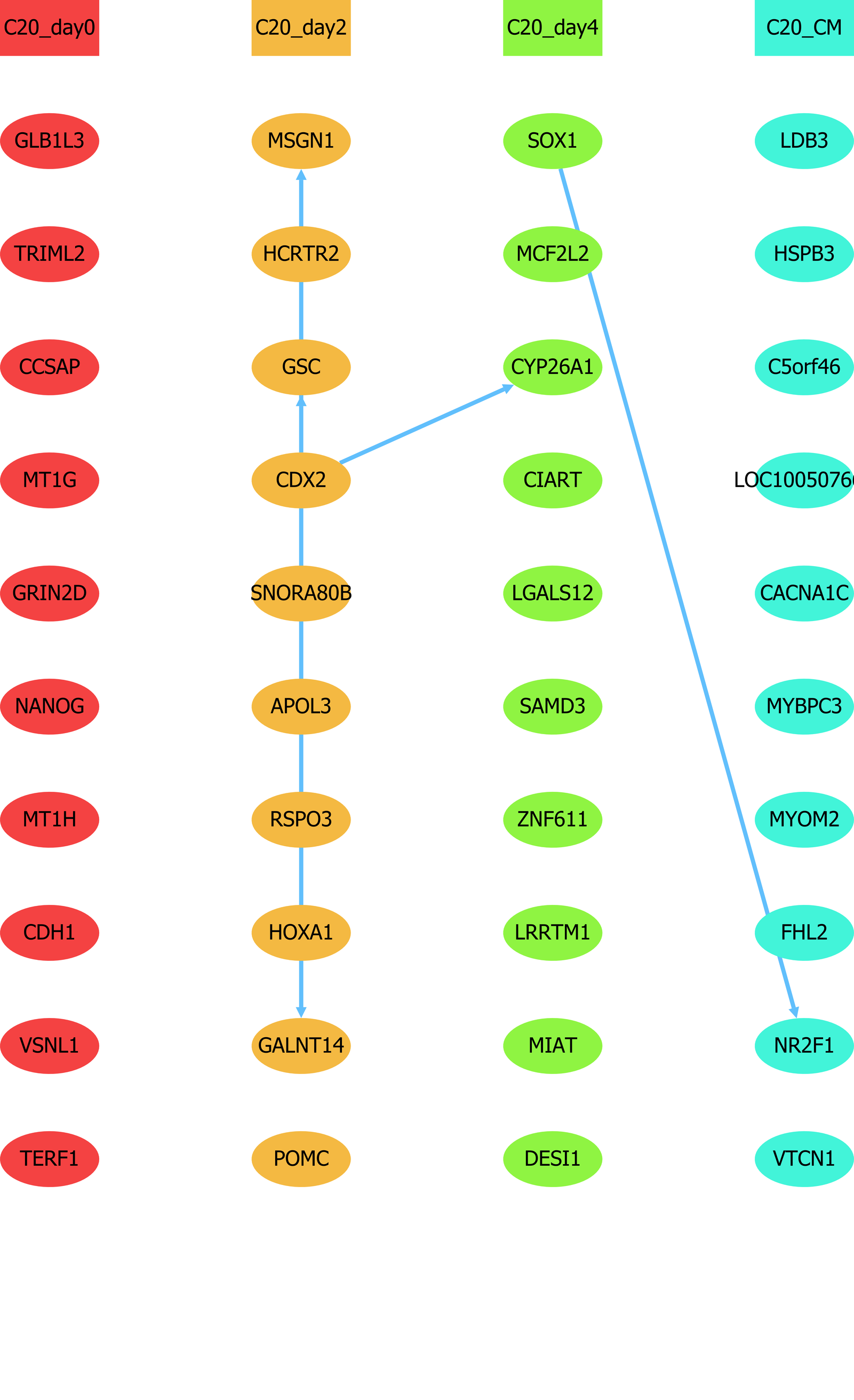

Supplement: S1 Fig — Stage-specific gene sets are not restricted to regulators in this example. This allows the TRC to include peaking non regulatory genes as well. (TIF) [file pone.0231326.s006.tif]
